# Disruption in Connexin-Based Communication Is Associated with Intracellular Ca^2+^ Signal Alterations in Astrocytes from Niemann-Pick Type C Mice

**DOI:** 10.1371/journal.pone.0071361

**Published:** 2013-08-15

**Authors:** Pablo J. Sáez, Juan A. Orellana, Natalia Vega-Riveros, Vania A. Figueroa, Diego E. Hernández, Juan F. Castro, Andrés D. Klein, Jean X. Jiang, Silvana Zanlungo, Juan C. Sáez

**Affiliations:** 1 Departamento de Fisiología, Pontificia Universidad Católica de Chile, Santiago, Chile; 2 Departamento de Neurología, Facultad de Medicina, Pontificia Universidad Católica de Chile, Santiago, Chile; 3 Instituto Milenio, Centro Interdisciplinario de Neurociencias de Valparaíso, Valparaíso, Chile; 4 Departamento de Gastroenterología, Facultad de Medicina, Pontificia Universidad Católica de Chile, Santiago, Chile; 5 Department of Biochemistry, University of Texas Health Science Center, San Antonio, Texas, United States of America; 6 FONDAP Center for Genome Regulation (CGR), Santiago, Chile; Albert Einstein College of Medicine, United States of America

## Abstract

Reduced astrocytic gap junctional communication and enhanced hemichannel activity were recently shown to increase astroglial and neuronal vulnerability to neuroinflammation. Moreover, increasing evidence suggests that neuroinflammation plays a pivotal role in the development of Niemann-Pick type C (NPC) disease, an autosomal lethal neurodegenerative disorder that is mainly caused by mutations in the *NPC1* gene. Therefore, we investigated whether the lack of NPC1 expression in murine astrocytes affects the functional state of gap junction channels and hemichannels. Cultured cortical astrocytes of NPC1 knock-out mice (Npc1^−/−^) showed reduced intercellular communication via gap junctions and increased hemichannel activity. Similarly, astrocytes of newborn Npc1^−/−^ hippocampal slices presented high hemichannel activity, which was completely abrogated by connexin 43 hemichannel blockers and was resistant to inhibitors of pannexin 1 hemichannels. Npc1^−/−^ astrocytes also showed more intracellular Ca^2+^ signal oscillations mediated by functional connexin 43 hemichannels and P2Y_1_ receptors. Therefore, Npc1^−/−^ astrocytes present features of connexin based channels compatible with those of reactive astrocytes and hemichannels might be a novel therapeutic target to reduce neuroinflammation in NPC disease.

## Introduction

Niemann-Pick type C (NPC) disease is an autosomal recessive neurodegenerative disorder that is caused by mutations in the *NPC1* or *NPC2* genes [1]. Most cases of NPC disease are caused by mutations in the *NPC 1* gene that yields a dysfunctional protein [1], [2]. NPC1 and NPC2 proteins are required for the trafficking of cholesterol; hence, a loss of function in these proteins results in the intracellular accumulation of free cholesterol and other lipids in late endosomes/lysosomes [3]. Progressive neurodegeneration, hepatosplenomegaly, and dysfunction of other organs are observed in patients affected with NPC disease [2]. These symptoms are also observed in a murine model of NPC disease [2], [4].

Npc1^−/−^ mice show hippocampal and cortical neuronal dysfunction [5]–[7], apoptosis of Purkinje neurons of the cerebellum and neuronal death in different brain regions [8]–[10]. Astrocytes express NPC1; and in the Npc1^−/−^ mouse brain, Npc1^−/−^ astrocytes exhibit morphological changes and become activated [11], [12]. The global neuronal deletion of NPC1, but not astrocyte-specific NPC1 deficiency, leads to the complete development of NPC neuropathology [13], which suggests that neuronal NPC1 deficiency is sufficient to mediate neurodegeneration. However, rescuing NPC1 expression in astrocytes delays neuronal loss and prolongs the life span in Npc1^−/−^ mice [14], suggesting that astrocytes may play an important role in the neuroinflammatory state of NPC disease. Neuroinflammation is present in Npc1^−/−^ mouse brain at an early post-natal age and is characterized by an enhanced number of microglia, increased levels of interleukin-1β and the presence of activated astrocytes [15]. Because astrocytes form extensive communicating networks [16], it is conceivable that NPC-induced neurodegeneration could depend on intercellular signaling and coordination among astrocytes. Such intercellular communication between astrocytes is partially attained by sharing cytoplasmic content through gap junction channels (GJCs); these intercellular channels allow direct but selective cytoplasmic communication between contacting cells, thereby promoting the exchange of metabolites and second messengers [17]. Each GJC is formed by the serial docking of two hemichannels (HCs), each contributed by one of two adjacent cells. HCs are composed of six protein subunits termed connexins (Cxs) [18]. Under defined conditions HCs mediate the uptake or release of ions and small molecules such as Ca^2+^ and ATP, respectively [19]. *In vivo*, astrocytes express Cxs 26, 30 and 43; *in vitro*, however primary cultures express mainly Cx43, which forms GJCs and HCs [20]. Interestingly, increasing evidence indicates that under pro-inflammatory conditions, astrocytes exhibit decreased GJC-mediated intercellular communication and increased HC activity [21]–[24]. Consequently, it has been proposed that these changes could be critical in initiating and maintaining the homeostatic imbalances present in diverse brain diseases [18]. Therefore, we examined whether NPC1 deficiency could affect the functional state of GJCs and HCs in astrocytes. Here, Npc1^−/−^ astrocytes were found to exhibit decreased gap junctional communication and increased HC activity. Importantly, the increased Cx43 HC activity observed in Npc1^−/−^ astrocytes mediated intracellular Ca^2+^ signal oscillations that were P2Y_1_ receptor-dependent.

## Methods

### Reagents and Antibodies

Anti-rabbit IgG antibodies-conjugated to horseradish peroxidase (HRP) were purchased from Pierce (Rockford, IL, USA). HEPES, DMEM, DNAse I, water (W3500), ethidium (Etd) bromide, Lucifer yellow (LY), U18666A, LaCl_3_ (La^3+^), probenecid and filipin were purchased from Sigma-Aldrich (St. Louis, MO, USA). Fetal bovine serum was obtained from Hyclone (Logan, UT, USA). Penicillin, streptomycin, goat anti-mouse Alexa Fluor 488 and goat anti-mouse Alexa Fluor 555 were obtained from Invitrogen (Carlsbad, CA, USA). Anti-GFAP monoclonal antibody was purchased from ICN Chemicals, (Irvine, CA). Anti-Cx43 monoclonal antibody was obtained from BD Biosciences (Franklin Lakes, NJ). Normal goat serum (NGS) was purchased from Zymed (San Francisco, CA, USA). Adenosine-5′-triphosphate disodium (Na_2_ATP) was from Roche Applied Science (Mannheim, Germany). The P2 antagonists 2′-Deoxy-N6-methyladenosine 3′,5′-bisphosphate tetrasodium salt (MRS2179) and N-[1-[[(Cyanoamino)(5-quinolinylamino) methylene]amino]-2,2-dimethylpropyl]-3,4-dimethoxy benzeneacetamide (A7400031) were from Tocris (Bristol, UK). Fura-2 AM was obtained from Molecular Probes (Eugene, Oregon, USA). A previously described rabbit polyclonal antibody (Cx43^(E2)^) against the second extracellular loop of Cx43, was used to specifically block Cx43 HCs [25].

### Animals

This study was carried out in strict accordance with the recommendations in the Guide for the Care and Use of Laboratory Animals of the National Institutes of Health (NIH). The protocol also followed local guidance documents generated by the ad hoc committee of the Chilean (CONICYT) and was approved by the Bioethics Committee of the School of Medicine and the Biological Sciences Faculty from Pontificia Universidad Católica de Chile (Protocol number 0852009 and CEBA, respectively). For all experiments post-natal day 2 mice (42 pups) were rapidly decapitated with a sharp surgical knife and all efforts were made to minimize suffering. BALB/c mice carrying a heterozygous mutation in the *Npc1* gene were kindly provided by Dr. Peter Pentchev (U.S. National Institutes of Health, Bethesda, MD, USA). The genotypes of the mice (wild-type, Npc1^+/+^; heterozygous, Npc1^+/−^ or Npc1-deficient, Npc1^−/−^) were determined by polymerase chain reaction (PCR)–based screening, as described previously [26].

### Cell Cultures

Astrocytes were prepared from the cortex of wild type or Npc1^−/−^ mice at post-natal day 2 as described [21]. Briefly, dissected meninges were carefully peeled off and cortices were mechanically dissociated. Cells were seeded onto 35-mm plastic dishes (Nunclon, Roskilde, Denmark) or onto glass coverslips (Gassalem, Limeil-Brevannes, France) placed inside 16-mm 24-well plastic plates (Nunclon) at a density of 5×10^5^ cells/dish or 1×10^5^ cells/well, respectively. Cells were cultured in DMEM, supplemented with penicillin (5 U/ml), streptomycin (5 µg/ml) and 10% fetal bovine serum. After 8 to 10 days, 1 µM cytosine-arabinoside was added for 3 days to eliminate proliferating microglial cells. Medium was changed twice per week, and the cultures were used after 3 weeks. These cultures contained >95% GFAP^+^ cells.

### Acute Hippocampal Slices

Acute transverse hippocampal slices (300–400 µm) were prepared from post-natal day 2 Npc1^+/+^ and Npc1^−/−^ mice (4 animals of each phenotype). Following decapitation of mice, their brains were dissected and placed in ice-cold artificial CSF (ACSF) containing the following (in mM): 125 NaCl, 2.5 KCl, 25 glucose, 25 NaHCO_3_, 1.25 NaH_2_PO_4_, 2 CaCl_2_, and 1 MgCl_2_, bubbled with 95% O_2_/5% CO_2_, pH 7.4. Hippocampal coronal brain sections were cut using a vibratome (Leica, VT 1000GS; Leica, Wetzlar, Germany) filled with ice-cold ACSF. The slices were transferred at room temperature (20–22°C) to a holding chamber and immersed in oxygenated ACSF, pH 7.4, for a stabilization period of 1 h before use.

### Dye Transfer

Cells plated on glass coverslips were bathed with recording medium (HCO_3_^−^-free F-12 medium buffered with 10 mM HEPES, pH 7.2). The permeability mediated by gap junctions was tested by evaluating the transfer of LY that was microinjected into one cell to neighboring cells, as described previously [22], [27]. The incidence of dye coupling (I.D.C.) was scored as the percentage of injections that resulted in dye transfer from the injected cell to more than one neighboring cell. The coupling index was calculated as the mean number of cells to which the dye spread in positive cases. In all experiments, dye coupling was tested by injecting a minimum of 10 cells.

### Dye uptake and Time-lapse Fluorescence Imaging

#### Cell cultures

For time-lapse fluorescence imaging, astrocytes plated on glass coverslips were washed twice and were then exposed to Locke’s solution (containing: 154 mM NaCl, 5.4 mM KCl, 2.3 mM CaCl_2_ and 5 mM HEPES at pH 7.4) with 5 µM Etd. Fluorescence intensity was recorded in selected cells with ROIs (regions of interest) in their nuclei. Images were captured every 30 s using a Q Imaging model Retiga 13001 fast-cooled monochromatic digital camera (12-bit) (Qimaging, Burnaby, BC, Canada) in an Olympus BX 51W1I microscope. Metafluor software (version 6.2R5, Universal Imaging Co., Downingtown, PA, USA) was used for off-line image analysis and fluorescence quantification. To test for changes in slope, regression lines were fitted to points before and after various treatments using the Excel program. The mean values of the slopes were compared using GraphPad Prism software and expressed as AU/min. La^3+^ (200 µM) and Cx43^(E2)^ antibody (1∶500) were applied acutely or preincubated for 30 min, respectively.

#### Acute slices

For “snapshot” experiments, acute slices were incubated with 20 µM Etd for 5 min in a chamber with oxygenated (95% O_2_ and 5% CO_2_) ACSF, pH 7.4. They were then washed five times for 2 min each with ACSF, followed by fixation at room temperature with 2% paraformaldehyde for 30 min. Finally, the slices were mounted in Fluoromount G and examined using a confocal laser-scanning microscope (Olympus, Fluoview FV1000, Tokyo, Japan). The dye uptake ratio was calculated using ImageJ (NIH, USA) as the subtraction (F-F0) between the fluorescence (F) from representative cells (∼20 cells per slice field) and the background fluorescence (F0) measured where no labeled cells were detected. At least five fields were selected in each slice.

### Ca^2+^ Signal Imaging

Cells plated on glass coverslips were loaded with 5 µM Fura-2 AM in DMEM without serum for 30 min at 37°C. The coverslips were washed three times with Locke’s solution, followed by a de-esterification period of 10 min at 37°C. The experimental protocol for Ca^2+^ imaging involved data acquisition every 3 s (emission at 510 nm) at 340- and 380-nm excitation wavelengths using an Olympus BX 51W1I upright microscope with a 40×water immersion objective. Changes were monitored using an imaging system equipped with a Retga 1300I fast-cooled monochromatic digital camera (12-bit) (Qimaging, Burnaby, BC, Canada), a monochromator for fluorophore excitation, and the METAFLUOR software (Universal Imaging, Downingtown, PA) for image acquisition and analysis. Analysis involved the quantification of the number of pixels assigned to each cell. The average pixel value allocated to each cell was obtained from excitation at each wavelength and corrected for background. Due to the low excitation intensity, no bleaching was observed, even when cells were illuminated for a few minutes. The ratio was obtained by dividing the 340-nm fluorescence image by the 380-nm image on a pixel-by-pixel base (R = F340 nm/F380 nm).

### Immunofluorescence and Confocal Microscopy

For all immunostaining experiments, astrocytes grown on coverslips were fixed at room temperature in 2% paraformaldehyde for 30 min, washed three times with PBS, incubated in 0.1 M PBS-glycine three times for 5 min each, and rinsed in 0.1% PBS-Triton X-100 containing 10% NGS for 30 min. We first incubated cells for 2 h at room temperature with anti-GFAP polyclonal antibody (IgG1, 1∶500) diluted in 0.1% PBS-Triton X-100 with 2% NGS. After three rinses in 0.1% PBS-Triton X-100, the cells were incubated for 50 min at room temperature with goat anti-rabbit Alexa Fluor 555 (1∶1500). For Cx43 detection, cells were incubated with the anti-Cx43 monoclonal antibody (1∶500) for 1 h at room temperature. After three washes, cells were incubated for 50 min at room temperature with goat anti-mouse Alexa Fluor 488. After several washes, coverslips were mounted in Fluoromount G and examined using an upright microscope equipped with epifluorescence (Eclipse E800, Nikon). To visualize double immunostaining, a confocal laser-scanning microscope (Olympus, Fluoview FV1000, Tokyo, Japan) was used. The immunofluorescence images were analyzed using ImageJ. The images were obtained as a stack in which each image had an optical thickness of 1 µm; each image was rendered in gray scale and digitized. Afterwards, the ImageJ feature for analyzing particles was used, and the Feret’s diameters were measured to quantify the particle diameters of Cx43 immunoreactivity. Feret’s diameter is the measured distance between parallel lines that are tangent to an object’s profile and perpendicular to the ocular scale. Therefore, it is a measure of the greatest distance possible between any two points along the boundary of a ROI.

### Filipin Staining and U18666A Treatment

To cause cholesterol accumulation in late endosomes and lysosomes astrocytes were treated with 0.5 or 1 µg/ml of U18666A for 24 or 48 h. For filipin staining, astrocytes were fixed for 20 min with cold paraformaldehyde (4%) in PBS, rinsed twice with cold PBS, incubated for 20 min in glycine (20 mM, pH 7.4) and rinsed three times with PBS. After fixation, cells were permeabilized for 30 min with saponin (0.2%) and BSA (3%) in PBS, incubated for 2 h with filipin (25 µg/ml) at room temperature, rinsed with PBS and mounted in Fluoromont G. The samples were analyzed in an Olympus BX 51W1I microscope.

### Cell Surface Biotinylation and Quantification

Cells cultured on 100-mm dishes were washed three times with ice-cold Hank’s saline solution (pH 8.0), and 3 ml of sulfo-NHS-SS-biotin solution (0.5 mg/ml) was added, followed by incubation for 30 min at 4°C. Cells were washed three times with ice-cold saline containing 15 mM glycine (pH 8.0) to block unreacted biotin. The cells were harvested with a cocktail of protease and phosphatase inhibitors (100 mM Na_2_P_2_O_7_×10 H_2_O, 100 mM NaF, 200µg/ml trypsin inhibitor from soybean, 6.4 mM benzamidine, 7.6 mM ε-aminocaproic acid, 20 mM EDTA, 3.2 mM phenylmethanesulfonyl fluoride, 6.1 µM aprotinin, 20 µM leupeptin) and were incubated with an excess of immobilized NeutrAvidin (1 ml of NeutrAvidin per 3 mg of biotinylated protein). After incubating cells for 1 h at 4°C with this cocktail, 1 ml of wash buffer (saline solution, pH 7.2 containing 0.1% SDS and 1% Nonidet P-40) was added. The mixture was centrifuged for 2 min at 14,000 rpm at 4°C. The supernatant was removed and discarded, and the pellet was resuspended in 40 µl of saline solution at pH 2.8, which contained 0.1 M glycine to release the proteins from the biotin. After the mixture was centrifuged at 14,000 rpm for 2 min at 4°C, the supernatant was collected, and the pH was adjusted immediately by adding 10 µl of 1 M Tris at pH 7.5. The relative protein levels were measured using Western blot analysis as described below. The resulting immunoblot signals were scanned, and the densitometry was performed using ImageJ software. Densitometry units were normalized to the signal obtained from the total protein, which was measured using Ponceau red.

### Western Blot Analysis

Cell cultures were rinsed twice with PBS (pH 7.4) and harvested by scraping with a rubber policeman in ice-cold PBS containing protease and phosphatase inhibitors (see above). Proteins were measured in aliquots of cell lysates with the Bio-Rad protein assay (Bio- Rad, Richmond, CA, USA). Pelleted cells were resuspended in 40 µl of the protease and phosphatase inhibitor solution, placed on ice, and lysed by sonication. Aliquots of cell lysates or biotinylated cell surface proteins were resuspended in 1 X Laemli’s sample buffer, boiled for 5 min, separated on 8% SDS-PAGE and electro-transferred to nitrocellulose sheets. Nonspecific protein binding was blocked by the incubation of nitrocellulose sheets in PBS-BLOTTO (5% nonfat milk in PBS). After 30 min, blots were incubated with primary antibody for 1 h at room temperature or overnight at 4°C, followed by four 10-min PBS washes. Blots were incubated with a goat anti-rabbit antibody conjugated to HRP. Immunoreactivity was detected by enhanced chemiluminescence (ECL) using the SuperSignal kit (Pierce, Rockford, IL) according to the manufactureŕs instructions.

### Data Analysis

Most analyses and graphs were made using Microsoft Office Excel Professional Plus 2010 by Microsoft (Redmond, WA, USA). For figure composition and statistical analyses, the Prism 5.0 software by GraphPad Prism software Inc. was used (La Jolla, CA, USA). For each data group, results were expressed as the mean ± standard error (S.E.), where n refers to the number of independent experiments. For statistical analysis, each treatment was compared with its respective control, and significance was determined using a one-way ANOVA. Any significant results were further assessed by using Dunn’s multiple comparison test.

## Results

### Npc1^−/−^ Cortical Astrocytes show Reduced Gap Junctional Communication

**Figure 1:**
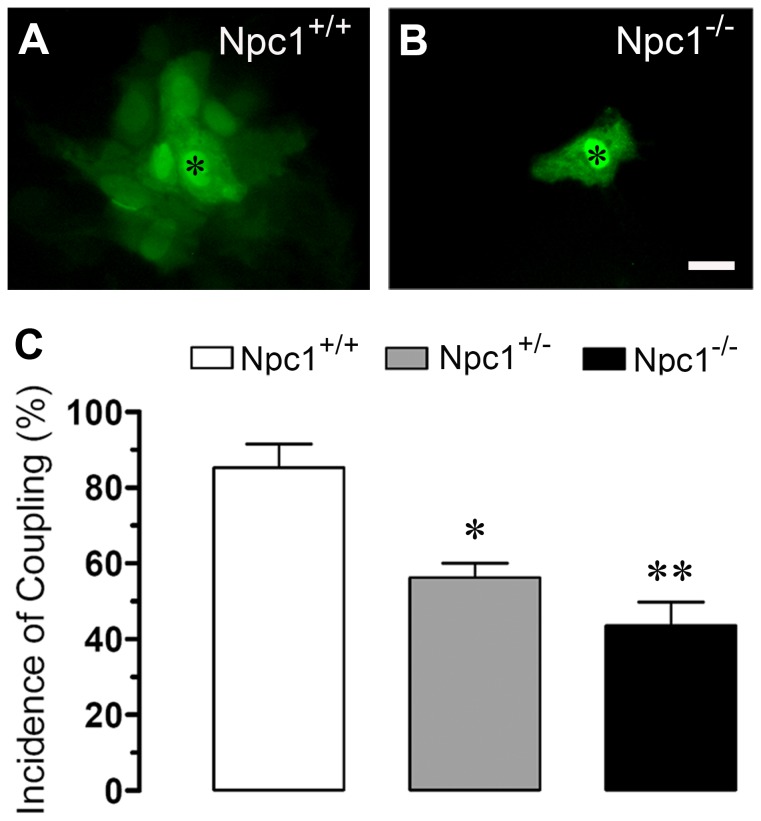
Npc1^−/−^ astrocytes exhibit reduced gap junctional communication. (A-B) Fluorescence micrographs of Lucifer yellow (LY) coupling in Npc1^+/+^ (A) and Npc1^−/−^ (B) astrocytes. The (*) in panels A-B denote the cell microinjected with LY. Calibration bar = 25 µm. (C) Average of incidence of coupling to LY in Npc1^+/+^ (white bar); Npc1^+/−^ (gray bar) and NPC1^−/−^ (black bar) astrocytes. *p<0.05 and ***p<0.005; compared to Npc1^+/+^ condition. The values are the means ± S.E. of 10 cells in a representative experiment of four separate cultures were used for each time point.

Several pro-inflammatory agents decrease the activity of GJCs in astrocytes, including cytokines, LPS and hypoxia-reoxygenation [23], [27]–[30]. In a similar manner, decreased gap junctional communication between astrocytes has been observed in different pathologies such as meningitis and diabetes [31], [32]. Therefore, we evaluated whether gap junctional communication was affected in Npc1^−/−^ cortical astrocytes. Using LY transfer, the I.D.C. was evaluated in cultured astrocytes. Npc1^−/−^ astrocytes exhibited a lower dye coupling (I.D.C.: 44±6%, n = 5) than Npc1^+/+^ astrocytes (85±6%, n = 4) (Figure 1A and B). Npc1^+/−^ astrocytes presented decreased coupling to an intermediate degree between Npc1^+/+^ and Npc1^−/−^ astrocytes (56±4%, n = 5) (Figure 1C); however, no significant differences in the index of coupling were found (Npc1^+/+^3.9±0.4, Npc1^+/−^3.7±0.4; Npc1^−/−^3.6±0.6), suggesting that some Npc1^−/−^ astrocytes remain in communication through GJCs.

### Increased Hemichannel Activity and Redox State in Npc1^−/−^ Cortical Astrocytes *in vitro*

**Figure 2:**
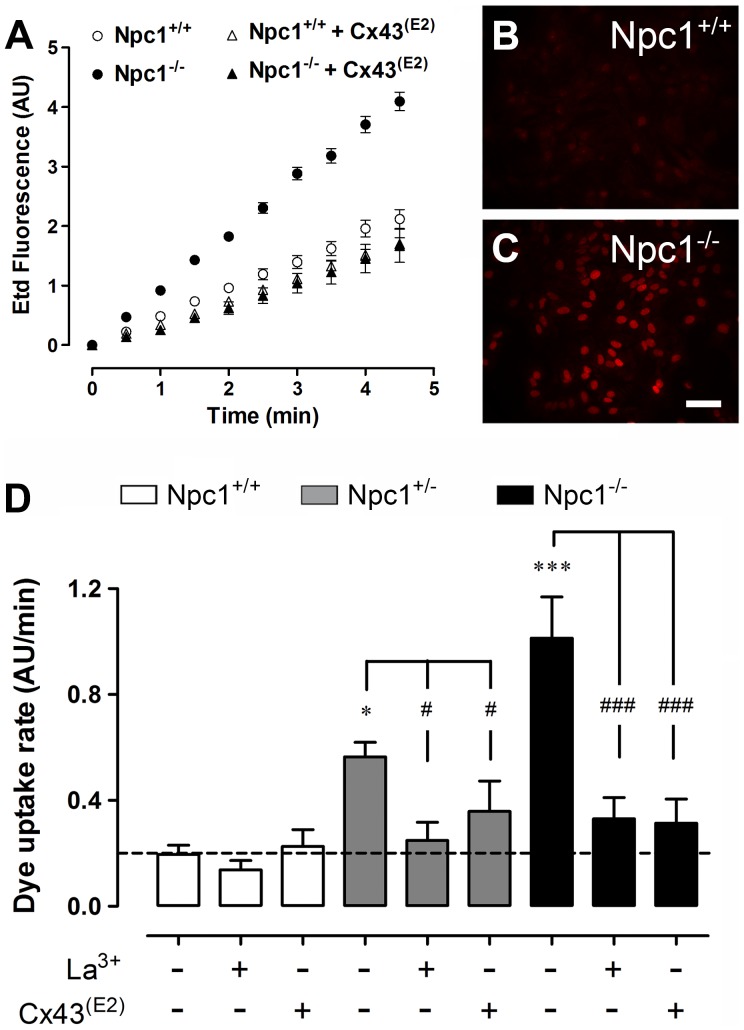
Npc1^−/−^ astrocytes exhibit increased Etd uptake mediated by Cx43 hemichannels. (**A**) Time-lapse measurements of ethidium (Etd) uptake in Npc1^+/+^ and Npc1^−/−^ (white and black circles, respectively) astrocytes under control conditions or pre-incubated for 15 min with the Cx43 HC blocking antibody Cx43^(E2)^ (white and black triangles, respectively). (**B–C**) Fluorescence micrographs of Etd uptake (5 min exposure to dye) in Npc1^+/+^ (**B**) and Npc1^−/−^ (**C**) astrocytes.(**D**) Etd uptake rate of Npc1^+/+^ (white bar); Npc1^+/−^ (gray bar) and NPC1^−/−^ (black bar) astrocytes under control conditions or treated with 200 µM La^3+^ (acutely added during experiment) or Cx43^(E2)^ antibody (pre-incubated 15 min before experiments). *p<0.05; **p<0.005 and ***p<0.001 compared to Npc1^+/+^ astrocytes. Each value corresponds to the mean ± S.E. of 20 cells in a representative of five experiments. Calibration bar = 60 µm.

NPC disease is associated with neuroinflammation [15], [33]. Because astroglial HC activity is increased by pro-inflammatory agents, including cytokines [23] and amyloid-β peptide [22], [27], the activity of HCs located at the cell surface was evaluated using time-lapse measurements of ethidium (Etd) uptake. Under control conditions, Npc1^+/+^ astrocytes showed a low Etd uptake rate (0.19±0.03 AU/min, n = 7); in contrast, both Npc1^+/−^ (0.56±0.06 AU/min, n = 7) and Npc1^−/−^ astrocytes (1.01±0.16 AU/min, n = 7) showed a proportionally higher Etd uptake (Figure 2A–D). Cx43 is the main Cx expressed in cultured cortical astrocytes [34], [35]; thus, to determine the contribution of Cx43 HCs in the increased Etd uptake, the astrocytes were acutely treated with La^3+^, a general blocker of Cx HCs [36]. The acute application of 200 µM La^3+^ decreased the Etd uptake rate in Npc1^+/−^ (0.25±0.07 AU/min, n = 5) and Npc1^−/−^ astrocytes (0.33±0.08 AU/min, n = 5) to values close to those observed in Npc1^+/+^ astrocytes (0.14±0.04 AU/min, n = 5) (Figure 2D).

To fully determine the contribution of Cx43 HCs in the above mentioned response, we used the Cx43^(E2)^ antibody that specifically blocks Cx43 HCs [25]. As with La^3+^, Cx43^(E2)^ antibody reduced the Etd uptake rate in Npc1^+/−^ (0.35±0.11 AU/min, n = 5) and Npc1^−/−^ astrocytes (0.31±0.09 AU/min, n = 5) to levels observed in Npc1^+/+^ astrocytes (0.22±0.06 AU/min, n = 5; Figure 2A and D). These results suggest that Cx43 HCs contribute to the increased Etd uptake observed in Npc1^−/−^ astrocytes.

Another gene family encoding a set of three membrane proteins, named pannexins (Panxs1–3), has recently been demonstrated to form HCs, which are activated by extracellular ATP via purinergic P2 receptors [37]. Accordingly, P2X_7_ receptor-induced dye uptake and ATP release through Panx1 HCs has been found in cortical astrocytes [37], [38]; however, experiments designed to evaluate Etd uptake and Ca^2+^ signaling mediated by P2X_7_ receptors and Panx1 HCs provided negative results. In these experiments, the extracellular addition of 300 µM ATP did not affect the Etd uptake rate in Npc1^+/+^, Npc1^+/−^ or Npc1^−/−^ astrocytes (Figure S1A and B); however, ATP induced a strong and similar peak in Ca^2+^ signal in Npc1^+/+^, Npc1^+/−^ and Npc1^−/−^ astrocytes (peak of 340/380 ratio: Npc1^+/+^0.35±0.02 AU; Npc1^+/−^0.34±0.02 AU; Npc1^−/−^0.39±0.02 AU, n = 4) (Figure S1C and D). Moreover, an increased Etd uptake rate in Npc1^−/−^ astrocytes was not affected by 1 mM probenecid, a Panx1 HC blocker (1.03±0.09 AU, n = 3, data not shown). Accordingly, immunofluorescence and Western blot analyses showed no changes in the Panx1 cellular distribution or total protein levels in Npc1^−/−^ astrocytes (n = 3 for each assay, data not shown). Altogether, these data suggest that Cx43 HCs are the major contributor to the increased Etd uptake observed in cultured Npc1^−/−^ astrocytes.

To explore the possible mechanisms involved in the Cx43 HC-mediated increase in Etd uptake of Npc1^−/−^ astrocytes we evaluated the effect of a reducing agent on Cx43 HC activity. Previously, we demonstrated that astrocytes under pro-inflammatory conditions present an oxidized state that activates Cx43 HCs, whereas in normal astrocytes a reducing agent increases the activity of Cx43 HCs [23], [39]. Oxidative stress damage and neuroinflammation have been demonstrated in NPC disease [8], [40]–[43]. Thus, we studied the effect of dithiothreitol (DTT), a –SH group reducing agent, on the activity of Cx43 HCs of different Npc astrocytes. After the application of 10 mM DTT, the Etd uptake rate was partially reduced in Npc1^−/−^ (0.63±0.16 AU/min, n = 4), not affected in Npc1^+/−^ (0.53±0.01 AU/min, n = 4), and increased in Npc1^+/+^ astrocytes (0.34±0.08 AU/min, n = 5) (data not shown). These data suggest that Cx43 HCs are reduced in Npc1^+/+^ astrocytes, whereas they are oxidized and in an intermediate redox state in Npc1^−/−^ Npc1^+/−^ astrocytes, respectively. To evaluate the astrocyte HC activity *in vitro* in another NPC model, we treated Npc1^+/+^ astrocytes with U18666A, a well-known NPC cellular phenotype-inducer [44]. As expected, Npc1^+/+^ astrocytes treated with U18666A (0.5–1 µg/ml) exhibited an increase in filipin staining that was concentration- and time-dependent (Figure S2A). Indeed, Npc1^+/+^ astrocytes treated with 1 µg/ml U18666A for 48 h showed the same high filipin staining observed in Npc1^−/−^ astrocytes (Figure S2A). The Etd uptake was also evaluated using cultured astrocytes under similar conditions and no significant changes in Etd uptake were observed in Npc1^+/+^ astrocytes treated with 0.5 µg/ml U18666A for 24 h (133±26% of control, n = 4) or 48 h (129±14% of control, n = 4). In addition, no changes in Etd uptake were observed in Npc1^+/+^ astrocytes treated for 24 or 48 h with the vehicle (ethanol). However, a slight increase in Etd uptake was found in Npc1^+/+^ astrocytes treated with 1 µg/ml U18666A for 24 h (176±23% of control, n = 4) or 48 h (184±15% of control, n = 4). The U18666A-induced Etd uptake in Npc1^+/+^ astrocytes was completely abrogated by 200 µM La^3+^, which was acutely applied after 24 (110±14% of control, n = 4) or 48 h (106±16% of control, n = 4) treatment with 1 µg/ml U18666A (Figure S2B and C). These results suggest that the U18666A-induced Etd uptake only partially mimics the increased Etd uptake of Npc1^−/−^ astrocytes.

### Npc1^−/−^ Astrocytes show Increased HC Activity in Acute Hippocampal Slices

**Figure 3:**
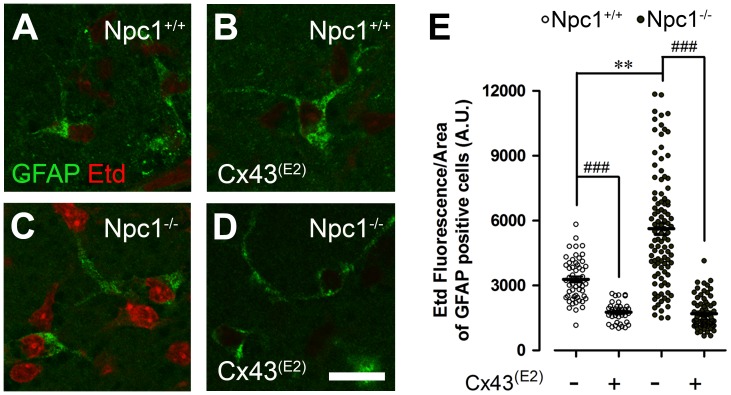
Astrocytes of hippocampal slices from Npc1^−/−^ mice exhibit an increased Cx43 hemichannel activity. **(A–D)** Representative images of ethidium (Etd, red) uptake by GFAP astrocytes (green) in hippocampal slices from Npc1^+/+^ (**A–B**) and Npc1^−/−^ (**C–D**) mice under control conditions (**A** and **C**) or pre-incubated for 15 min with the Cx43 HC blocking antibody Cx43^(E2)^ (**B** and **D**). Calibration bar = 20 µm. (**E**). Averaged data of Etd uptake of hippocampal Npc1^+/+^ and Npc1^−/−^ astrocytes (white and black circles, respectively) under control conditions or pre-incubated for 15 min with Cx43^(E2)^ antibody. **p<0.005 compared to Npc1^+/+^ astrocytes. The averaged data were obtained from four independent experiments.

To evaluate whether the increased Etd uptake observed in cultured Npc1^−/−^ astrocytes could also occur in an integrated system, we evaluated the Etd uptake in acute hippocampal slices, as previously described [22]. Astrocytes were identified by their glial fibrillary acidic protein (GFAP) reactivity, and Etd uptake was evaluated in snapshot experiments after the fixation of hippocampal slices. Compared to GFAP positive cells in hippocampal slices from Npc1^+/+^ mice (3285±134 AU, n = 53), a significantly higher but heterogeneous Etd uptake was observed in many GFAP-positive cells in hippocampal slices from Npc1^−/−^ newborn mice (Etd uptake of GFAP positive cells: 5628±258 AU, n = 102) (Figure 3). The increased Etd uptake in Npc1^−/−^ GFAP-positive cells was completely abrogated with the application of Cx43^(E2)^ antibody (1695±79 AU, n = 77), and the Etd uptake was also significantly reduced in Npc1^+/+^ GFAP-positive cells (1764±68 AU, n = 42) (Figure 3). Thus, hippocampal Npc1^−/−^ astrocytes show increased Cx43 HC activity at very early stages of brain development.

### Npc1^−/−^ Astrocytes Present Increased P2Y_1_-dependent Ca^2+^ Signal Oscillations

**Figure 4:**
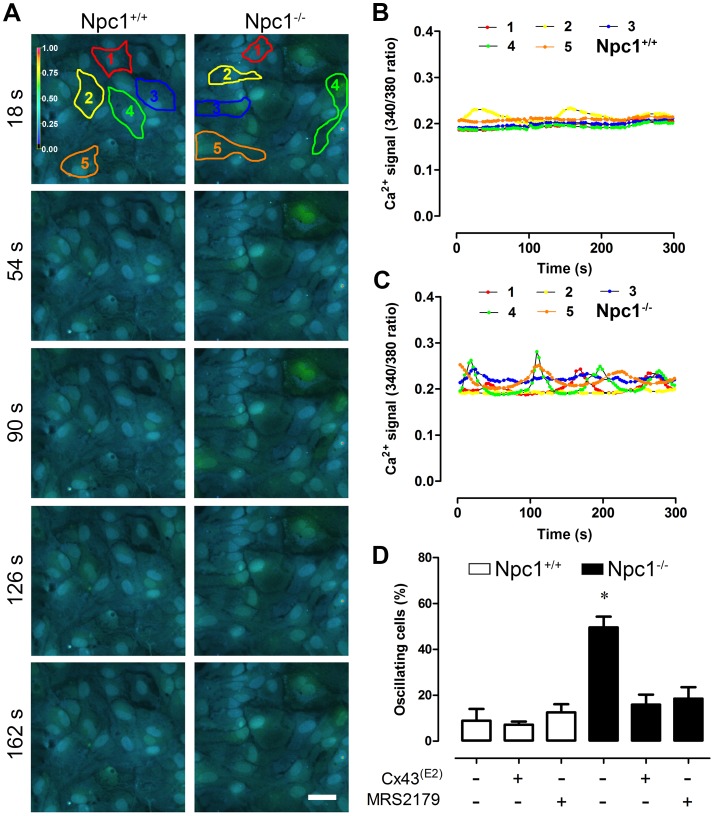
Npc1^−/−^ astrocytes exhibit increased Ca^2+^ signal oscillations mediated by Cx43 hemichannels and P2Y_1_ receptors. (**A**) Representative fluorescent micrographs of time-lapse imaging showing spontaneous changes in the Fura-2 ratio (pseudo-colored scale) in Npc1^+/+^ and Npc1^−/−^ astrocytes at the indicated times. Scale bar = 25 µm. (**B**–**C**) Plots of Ca^2+^ signals over time of cells 1 (red), 2 (yellow), 3 (green), 4 (orange) and 5 (blue) depicted in panel **A** for Npc1^+/+^ (**B**) and Npc1^−/−^ (**C**) astrocytes. (**D**) Averaged data of number of cells (%) exhibiting Ca^2+^ oscillations normalized to total cells per field in Npc1^+/+^ and Npc1^−/−^ (white and black bars, respectively) astrocytes under control conditions or pre-incubated with Cx43^(E2)^ antibody or the P2Y_1_ receptor blocker, MRS2179. *p<0.05. The averaged data were obtained from at least four independent experiments.

Several neurodegenerative diseases are characterized by distorted Ca^2+^ signaling [45]. Extracellular ATP and purinergic signaling play a pivotal role in Ca^2+^ signaling in astrocytes and contribute to the development of neuroinflammation in several neurodegenerative diseases [46]. ATP is released from astrocytes by several means, including Cx and Panx HCs [38], [47], which contributes to the amplification of extracellular Ca^2+^ waves [48], [49]. Given the increased HC activity observed in Npc1^−/−^ astrocytes, possible changes in intracellular Ca^2+^ signal in astrocytes were evaluated using Fura-2. Under control conditions, only a few astrocytes from Npc1^+/+^ mice showed Ca^2+^ signal oscillations (oscillating cells: 8.9±5.1%, n = 4) (Figure 4A and B, Movie S1). The number of astrocytes cultured from Npc1^+/−^ mice showing Ca^2+^ signal oscillations was only slightly higher (15.1±3.6%, n = 4), in contrast the number of oscillating cells was significantly higher in cultures of Npc1^−/−^ astrocytes (49.5±4.7%, n = 5) (Figure 4A and C, Movie S1). However, oscillating Npc1^+/+^, Npc1^+/−^ and Npc1^−/−^ astrocytes exhibited a similar number of individual oscillations (Npc1^+/+^2.1±0.2 AU, n = 34; Npc1^+/−^1.9±0.2 AU, n = 29; Npc1^−/−^2.1±0.1 AU, n = 79, data not shown). The contribution of Cx43 HCs to Ca^2+^ oscillations through ATP release has been observed in different cell types, including glia [48], [50]; thus, we evaluated their contribution in Ca^2+^ signal oscillations. Treatment with the Cx43^(E2)^ antibody reduced the number of oscillating cells in Npc1^−/−^ astrocytes (15.9±4.4%, n = 4) to values similar to those observed in Npc1^+/+^ astrocytes treated with this blocker (7.1±1.4%, n = 4; Figure 4D), thereby implicating the participation of Cx43 HCs in this phenomenon.

The functional expression of P2X_7_ receptors has also been correlated with neuroinflammation in neurodegenerative diseases, and it is accepted that these receptors are permeable to Ca^2+^ [51]. Accordingly, we tested their possible contribution to Ca^2+^ signal oscillations. Treatment with 10 µM A7400031, a specific P2X_7_ receptor inhibitor, did not affect the number of oscillating cells in either in Npc1^+/+^ or Npc1^−/−^ astrocytes (oscillating cells: Npc1^+/+^5.3±1.3%; Npc1^−/−^44.8±4.9%, n = 3, data not shown). In addition, the exposure of Npc1^+/+^ and Npc1^−/−^ astrocytes to a medium without extracellular Ca^2+^ did not affect the Ca^2+^ signal oscillations (oscillating cells: Npc1^+/+^4.8±1.4%; Npc1^−/−^42.6±8.3%, n = 3, data not shown). Therefore, it is unlikely that P2X_7_ receptors participate in the increased Ca^2+^ signal oscillations observed in Npc1^−/−^ astrocytes.

Astrocytes express several purinergic P2 receptors, including the P2Y_1_ receptors that play a pivotal role in the Ca^2+^ responses to synaptic activity [52], [53]. Interestingly, P2Y_1_ receptor expression in astrocytes is regulated by the carboxy-terminal domain of Cx43 [54]. Moreover, the link between Cx43 HCs, P2Y_1_ receptors, purinergic and Ca^2+^ signaling has been recently shown in tanycytes, another type of glia [50]. Thus, we decided to evaluate whether P2Y_1_ receptors are involved in the increased oscillations of Ca^2+^ signal observed in Npc1^−/−^ astrocytes. Incubation with 10 µM MRS2179, a specific P2Y_1_ receptor inhibitor, completely abrogated the increased number of Npc1^−/−^ astrocytes that showed Ca^2+^ oscillations (18.5±5.1%, n = 4), but did not affect the number of Ca^2+^ signal oscillations of Npc1^+/+^ astrocytes (12.5±3.6%, n = 4; Figure 4D). Altogether, these data suggest that the number of Npc1^−/−^ astrocytes that show Ca^2+^ signal oscillations depends on functional Cx43 HCs and P2Y_1_ receptors.

### Npc1^−/−^ Astrocytes Contain Large Cx43 Aggregates and Reduced Surface Cx43

**Figure 5:**
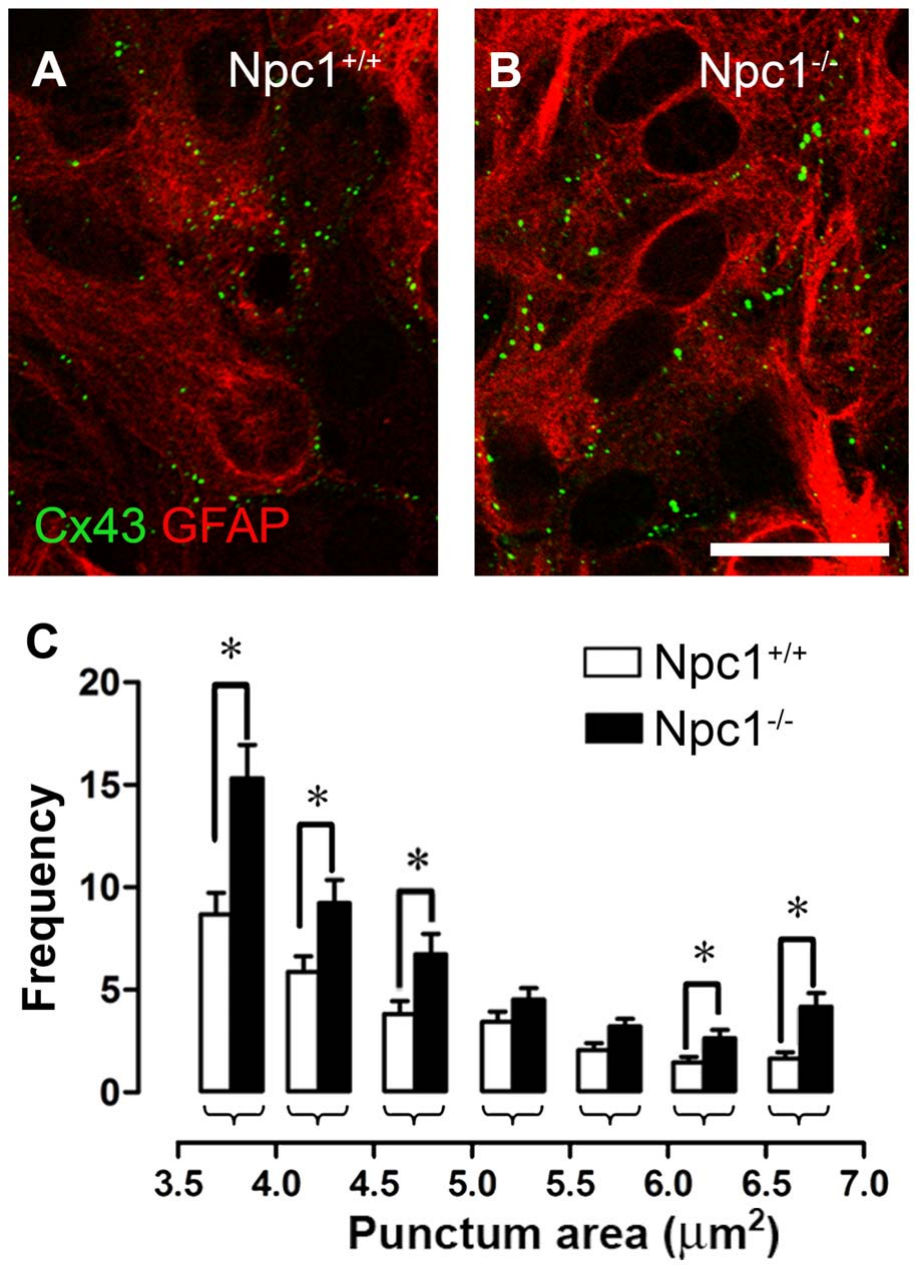
Npc1^−/−^ astrocytes exhibit larger Cx43 aggregates. (**A–B**) Representative confocal images depicting Cx43 (green) and GFAP (red) immunolabeling in Npc1^+/+^ (**A**) and Npc1^−/−^ (**B**) astrocytes. Bar = 15 µm. (**C**) Quantification of the frequency of Cx43 spots of different diameters in Npc1^+/+^ and Npc1^−/−^ (white and black bars, respectively) astrocytes. *p<0.05. Averaged data were obtained from at least three independent experiments.

In different pathophysiological conditions, astrocytes redistribute their Cx43 [21], [23], [30]. To evaluate possible changes in the cellular distribution of Cx43 in Npc1^−/−^ astrocytes, immunofluorescence was performed in GFAP positive cells. Npc1^−/−^ astrocytes showed heterogeneous Cx43 labeling, including vesicle-like structures or puncta of different shapes and sizes (Figure 5). The larger puncta might correspond to gap junction plaques or internalized gap junctions, and the smaller puncta might correspond to Cx43 positive trans-Golgi vesicles sorted to the plasma membrane or small junctions internalized and targeted for degradation [55], [56]. The quantification of punctate areas revealed no significant differences between Npc1^+/+^ and Npc1^−/−^ astrocytes for small or intermediate Cx43 puncta (0.5–3.5 µm^2^, not shown). However, Npc1^−/−^ astrocytes contained a higher frequency of large Cx43 puncta (3.8 µm^2^∶15.3±1.6; 4.3 µm^2^∶9.2±1.1; 4.8 µm^2^∶6.7±1.0; 6.2 µm^2^∶2.6±0.4; 6.7 µm^2^∶4.2±0.7; n = 4) than Npc1^+/+^ astrocytes (3.8 µm^2^∶8.7±1.0; 4.3 µm^2^∶5.9±0.8; 4.8 µm^2^∶3.8±0.6; 6.2 µm^2^∶1.5±0.3; 6.7 µm^2^∶1.6±0.3; n = 4) (Figure 5C). These reactive regions might correspond to large internalized gap junction plaques [55].

**Figure 6:**
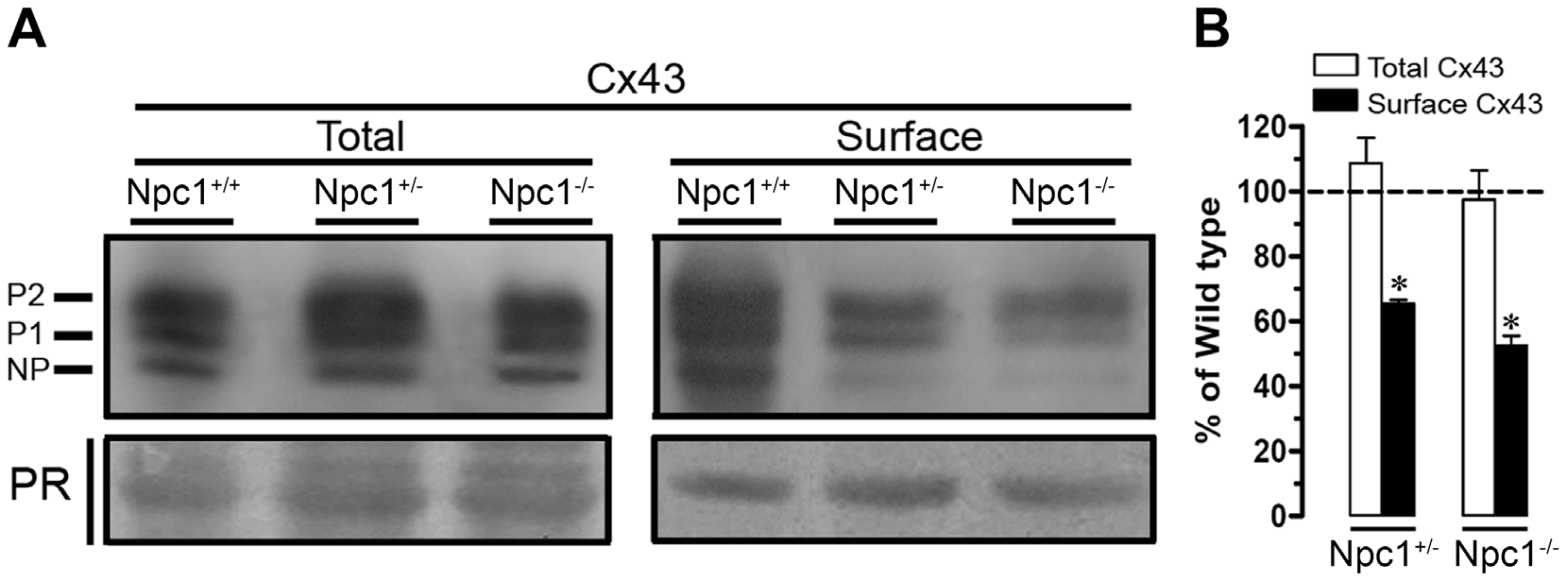
Lack of NPC1 protein reduces surface but not total levels of Cx43 in astrocytes. (**A**) Representative western blots of the relative levels of total Cx43 (left panel) and total Cx43 (right panel) present in cultures of Npc1^+/+^, Npc1^+/−^ and Npc1^−/−^ astrocytes. Ponceau red (PR) was used as a loading control. The phosphorylated (P1–P2) and non-phosphorylated (NP) forms of Cx43 are indicated on the left. (**B**) Quantification normalized to Npc1^+/+^ levels (wild type, dashed line) of total and surface (white and black bars, respectively) Cx43 levels in Npc1^+/−^ and Npc1^−/−^ astrocytes. Total and surface levels of Cx43 were normalized according to the PR levels detected in each lane. *p<0.05 compared to Npc1^+/+^. The averaged data were obtained from at least three independent experiments.

To test whether the lack of NPC1 protein in cultured astrocytes alters the surface or total levels of Cx43, biotinylation of cell surface proteins and Western blot analyses were performed. The total Cx43 levels were similar in Npc1^+/+^, Npc1^+/−^ and Npc1^−/−^ astrocytes (Figure 6). Nevertheless, surface levels of Cx43 were reduced in Npc1^+/−^ (65.5±1.8%; n = 3) or Npc1^−/−^ (52.5±5.3%; n = 3) astrocytes compared to Npc1^+/+^ astrocytes (Figure 6).

## Discussion

In this study, we demonstrated that Npc1^−/−^ astrocytes exhibit reduced gap junctional communication and increased membrane permeability, which contribute to Cx43 HC and P2Y_1_ receptor-dependent intracellular Ca^2+^ signal oscillations. These changes were only partially recapitulated by treating Npc1^+/+^ astrocytes with U18666A. Finally, Npc1^−/−^ astrocytes contained larger Cx43 immunoreactive aggregates (large puncta staining) lower Cx43 surface levels.

Here, we provide evidence that Npc1^−/−^ astrocytes exhibit a connexin-based channels activity with a “reactive” phenotype similar to that of astrocytes treated with pro-inflammatory cytokines [23]. This phenotype includes reduced gap junctional communication and increased HC activity. The reduction in astrocytic gap junctional communication is a hallmark of neuroinflammatory and neurodegenerative conditions [20], which *in vivo* might predispose the surrounding neurons to cell death due to the reduced spatial buffering mediated by astrocytes.

Further studies are required to determine the *in vivo* relevance of our findings. Several studies have shown that neuronal death is a cell-autonomous process in NPC disease [13], [57]–[59]. Our results suggest that apoptosis is the main way of neuron death in NPC disease [8], [60]. However, several studies have shown that increased lipid storage in NPC induces autophagy [57], [58], [61], [62], a cellular response that enables recycling of damaged organelles to promote cell survival [63]. Interestingly, the autophagic pathway is a source of cholesterol for the lysosome, and genetic or pharmacological inhibition of autophagy reduced cholesterol storage and lysosomal dysfunction in NPC cellular models [64]. Therefore, persistent activation of autophagy can lead to cell stress, suggesting that autophagy and apoptosis contribute to cell death and tissue degeneration in NPC disease. *In vivo* neuron specific Npc1 rescue is sufficient to prevent neuron degeneration and to ameliorate the disease in Npc1^−/−^ mice [58], [59]. Consequently, NPC1 deficiency in neurons is sufficient to mediate CNS disease [13]. In addition, NPC1 rescue in astrocytes did not prevent neurodegeneration or slow the progression of the disease [59], and NPC1 deficiency in astrocytes did not lead to CNS pathology in NPC1^−/−^ mice [13]. However, Zhang et al (2008) showed that an astrocyte-targeted GFAP promoter-driven NPC1 transgene can triple the life span of Npc1^−/−^ mice [14]. Moreover, recent evidence supports the notion that astrocytes play a pivotal role in NPC disease progression [65]. This work showed that simultaneous recovery of Npc1 in neurons and astrocytes decreased the rate/degree of decline in Npc1^−/−^ mice, compared to Npc1 recovery only in neurons [65]. In the same way, our results in astrocyte cultures show impairment in gap junctional communication that could contribute to NPC pathogenesis.

Using a specific Cx43 HC blocker (Cx43^(E2)^ antibody), we demonstrated that the HC activity found in normal and in Npc1^+/−^ or Npc1^−/−^ astrocytes corresponds to Cx43 HCs. Panx1 did not contribute to membrane permeability in either Npc1^+/+^ or Npc1^−/−^ astrocytes, in contrast to previous observations made in wild-type astrocytes [37], [38], [66]. This disagreement could be explained by several variations in animal maintenance and culture conditions; including feeding, timing of total culture previous to measurements and cell density.

We found that Npc1^+/−^ astrocytes present an intermediate reduction of gap junctional communication and increase in Cx43 HC activity as compared to Npc1^−/−^ astrocytes. These findings are in agreement with previous reports that have shown an intermediate or altered phenotype in Npc1^+/−^ compared to Npc1^+/+^ and Npc1^−/−^ mice [67]–[69]. For example, it has been shown that a decreased gene dosage of Npc1^+/−^ mice promotes weight gain [68], and accelerates accumulation of amyloid-β peptide in a model of Alzheimer disease [67]. Also, increased expression of caveolin-1 has been reported in Npc1^+/−^ liver homogenates and fibroblasts [70], [71]. Moreover, our group have shown that Npc1^+/−^ mice fed with a lithogenic diet present a decreased biliary cholesterol secretion and an intermediate phenotype compared to Npc1^−/−^ and Npc1^+/+^ mice [69].

Cholesterol has been shown to accumulate in GFAP positive astrocytes and hippocampal slices of Npc1^−/−^ murine brains [72], and deficient synaptic activity has been demonstrated in hippocampal neurons [6], [7]. At earliest stages of disease progression, we found increased Cx43 HC activity in astrocytes of acute hippocampal slices from Npc1^−/−^ mouse brains. Therefore, we propose that increased Cx43 HC activity is one of the earliest events during the development of neuroinflammation in NPC disease.

The contribution of inflammation to NPC pathology is still a topic of study. The presence of inflammatory markers has been widely reported and is detected even at postnatal ages in the brain of Npc1^−/−^ mice [15]. However, López et al (2012) described that the deletion of the macrophage chemokine CCL3 or the complement molecule C1q did not alter CNS pathology [59], [73], [74]. In addition, *in vivo* rescue and deletion experiments on the *Npc1* gene show that neurons are the most relevant cell type involved in the pathogenesis of the disease. Overall, the evidence suggests that inflammation is a consequence of the disease; targeting inflammation could be beneficial to reduce the deleterious progression of the disease. Accordingly, the use of non-steroidal anti-inflammatory drugs (NSAIDs) in Npc1^−/−^ mice significantly prolongs their survival and slows the onset of symptoms [43], [75], suggesting that unknown inflammatory mediators might be involved in NPC disease.

HCs allow Ca^2+^ and glucose uptake and ATP release in astrocytes; thus, the changes in intercellular communication in Npc1^−/−^ astrocytes could lead to changes in their metabolic status [23], [27], [76]. Amyloid-β peptide is up-regulated in Npc1^−/−^ mouse brains [72], and previous studies reported an increased HC activity in hippocampal slices after 3 h of treatment with amyloid-β peptide [22]. This increased HC activity leads to neuronal cell death in the hippocampus through the release of ATP and glutamate [22]. Consistent with the previous reports of increased hippocampal neuronal cell death in Npc1^−/−^ mice [77], we suggest a similar mechanism in which increased HC activity could lead to neuronal death.

Although the differential regulation of GJCs and HCs in Npc1^−/−^ astrocytes is similar to that observed in astrocytes under inflammatory conditions [23], Npc1^−/−^ astrocytes did not display a fully inflammatory phenotype. Indeed, DTT treatment completely abrogated HC activity of astrocytes treated with pro-inflammatory cytokines [23]. Even when oxidative stress has been demonstrated in NPC disease [8], [40], [41], treatment with DTT only partially reduced Etd uptake in Npc1^−/−^ astrocytes, suggesting that other mechanism could contribute to the increased HC activity. Alternatively, it is possible that the inflammatory phenotype of Npc1^−/−^ astrocytes partially recovers after several days in culture, but the astrocytes could be more affected by the redox state of the organ in the natural environment of the brain.

We also explored a pharmacological model of NPC disease using U18666A, which partially induces the NPC cellular phenotype. We observed a slight increase in the HC activity of Npc1^+/+^ astrocytes treated with U18666A, which caused cholesterol accumulation to a similar extent to NPC1^−/−^ astrocytes, as shown previously [78]. Nevertheless, the U18666A-induced HC activity did not completely mimic the increased Etd uptake of Npc1^−/−^ astrocytes, possibly because U18666A does not fully induce the NPC phenotype, as shown previously *in vivo* [79]. Although U18666A has been widely used to induce the NPC phenotype and leads to lipid accumulation in late endomes/lysosomes, it should be noted that this agent is toxic at high concentrations and prolonged incubation times [79]. In addition, U18666A also affects the activity of HMG-CoA reductase, the cholesterol synthesis rate-limiting enzyme, in a concentration-dependent manner [80]. Therefore, this pharmacological NPC model has some limitations. The notion that cholesterol accumulation itself disrupts neuronal viability is supported by recent observations, which show that using cyclodextrin to normalize cholesterol homeostasis delays neuronal death [81]. Interestingly, the U18666A-induced accumulation of cholesterol induces the release of TNF-α in macrophages [82], which increases the HC activity in astrocytes [23], and suggests that cholesterol accumulation might act as a pro-inflammatory factor. Further studies should be performed to determine whether cholesterol accumulation together with pro-inflammatory cytokines could fully recapitulate the Npc1^−/−^ phenotype.

As shown in several neurodegenerative disorders [45], NPC disease involves distorted Ca^2+^ signaling [83], [84]. Here, we demonstrated that inhibition of Cx43 HC abrogate the Ca^2+^ oscillations suggesting that Cx43 HCs contribute to increased purinergic signaling in Npc1^−/−^ astrocytes, likely through ATP release [47], [48], and not through Ca^2+^ influx because Ca^2+^ oscillations still occurred in the absence of extracellular Ca^2+^. The increased number of Ca^2+^ signal oscillations in Npc1^−/−^ astrocytes was also dependent on P2Y_1_ receptors. However, this increase did not depend on P2X_7_ receptors, as shown using a specific inhibitor. These observations corroborate recent reports of the interaction between Cx43 HCs and P2Y_1_ receptors in tanycytes [50]. Interestingly, previous reports showed that P2Y_1_ receptors are regulated by the carboxy-terminal domain of Cx43 [54]. A similar interaction might occur in Npc1^−/−^ astrocytes.

Cholesterol levels at the plasma membrane positively affect gap junctional communication [85]. However, in Npc1^+/−^ and Npc1^−/−^ astrocytes we found a reduction in intercellular communication. This suggests that NPC1 might regulate the transport of cholesterol to caveolin 1 and 2 compartments [70], [71], [86], which are known to regulate the trafficking of Cx43 and gap junctional communication [87]. The changes in the intracellular distribution of Cx43 found in Npc1^−/−^ astrocytes, which accumulate intracellular cholesterol, are consistent with a reduction in gap junctional communication. This redistribution of Cx43 could also explain why GJCs are down-regulated. This notion is further supported by the biotinylation analysis; low levels of Cx43 were found at the cell surface in Npc1^−/−^ astrocytes, similar to astrocytes under pro-inflammatory conditions [23]. This finding implies that the increase in Cx43 HC activity is due to the activation of a gating mechanism rather than to an increase in the number of surface Cx43 HCs.

**Figure 7:**
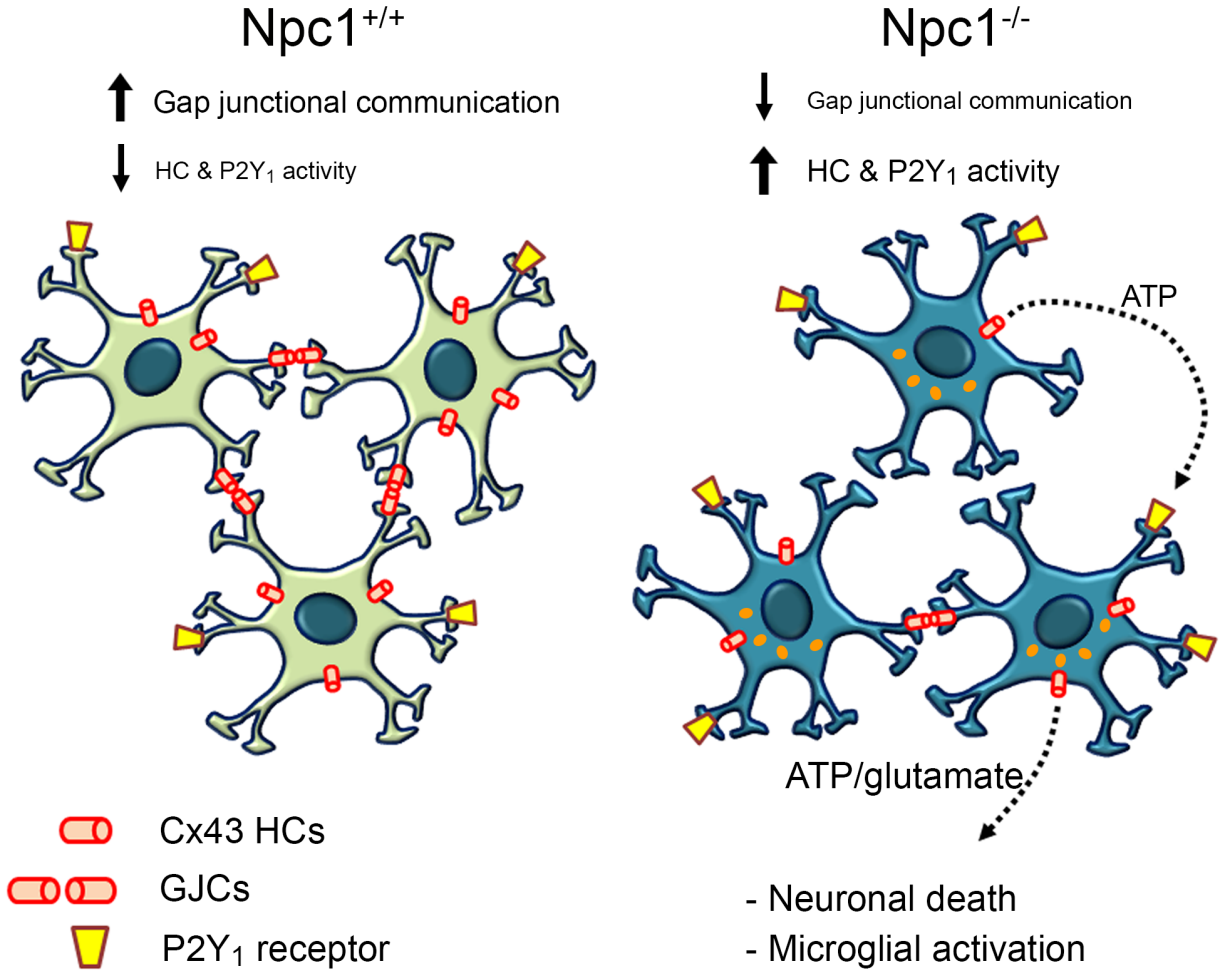
Model of role of Cx-based channels in NPC disease. Under resting conditions Npc1^+/+^ astrocytes exhibit high gap junctional communication and low HC activity. On the contrary, Npc1^−/−^ astrocytes under control conditions display cholesterol accumulation (orange droplets) and inflammatory-like phenotype with impaired gap junctional communication and increased HC activity. In addition, Npc1^−/−^ astrocytes have increase free intracellular Ca^2+^ signal oscillations mediated by the P2Y_1_ receptor. Altogether, these changes in astrocytic communication could lead to neuronal death and microglia activation, contributing to the neurodegeneration observed in NPC.

To summarize (Figure 7), an increase in the activity of Cx43 HCs in Npc1^−/−^ astrocytes could allow ATP and glutamate release. Consequently, Npc1^−/−^ astrocytes exhibit increased P2Y_1_ receptor activation, which could lead to neuronal death through microglia and astrocyte activation or direct neuroexcitotoxicity [27]. Although cultures of astrocytes and hippocampal slices do not fully recapitulate the mechanisms occurring in a NPC brain, we propose Cx HCs as a new therapeutic target for treating NPC disease. This novel pharmacological approaches could be used in combination with treatments to normalize cholesterol accumulation [81], thereby promoting neuronal survival.

## Supporting Information

Figure S1 Npc1^−/−^ astrocytes do not exhibit ATP-induced dye uptake. (A) Time-lapse measurements of Etd uptake in Npc1^+/+^ and Npc1^−/−^ astrocytes (white and black circles, respectively) exposed to 300 µM ATP. (B) Averaged data of Etd uptake rate of Npc1^+/+^, Npc1^+/−^ and Npc1^−/−^ astrocytes (white, grey and back bars, respectively) under control conditions or treated with 300 µM ATP (acutely added during experiment). No significant differences were observed after ATP treatment. (C-D) Representative plots of relative changes in the Ca^2+^ signal (340/380 ratio) over time in Npc1^+/+^ astrocytes (C, white circles) and Npc1^−/−^ astrocytes (D, black circles) under control conditions or after treatment with 300 µM ATP. In each panel, three photomicrographs of time-lapse images show changes in the Fura-2 ratio (pseudo-colored scale). Averaged data were obtained from at least three independent experiments. Each value corresponds to the mean ± S.E. of 20 cells in a representative of three experiments.
(TIF)

Figure S2 Cholesterol accumulation partially mimics the increased dye uptake of Npc1^−/−^ astrocytes. **(A)** Fluorescent micrographs of filipin staining (blue) in Npc1^+/+^, Npc1^+/−^ and Npc1^−/−^ astrocytes. Also shown fluorescent micrographs of filipin staining in Npc1^+/+^ astrocytes exposed to vehicle (EtOh) or treated with 0.5 or 1 µg/ml U1866A for 24 or 48 h. Calibration bar = 25 µm. (**B**-**C**) Averaged data (normalized to control; dashed line) of the rate of Etd uptake by Npc1^+/+^ astrocytes exposed to vehicle or 0.5 and 1 µg/ml U1866A for 24 (**B**) or 48 h (**C**). Additionally, the effect of 200 µM La^3+^ applied acutely during Etd uptake experiments is shown. *p<0.05 compared to the basal level of Npc1^+/+^ astrocytes. The averaged data were obtained from four independent experiments.
(TIF)

Movie S1 Time-lapse (4 min and 30 s) movie of spontaneous changes in the Fura-2 ratio (340/380 ratio, pseudo-colored scale) in Npc1^+/+^ and Npc1^−/−^ astrocytes (left and right, respectively). Frames in the movie were captured 3 s apart.
(AVI)
