# Supplementary figures and images for: Disruption in Connexin-Based Communication Is Associated with Intracellular Ca2+ Signal Alterations in Astrocytes from Niemann-Pick Type C Mice
Source: PLoS One. 2013 Aug 15;8(8):e71361. doi: 10.1371/journal.pone.0071361 (PMC3744576; doi:10.1371/journal.pone.0071361)

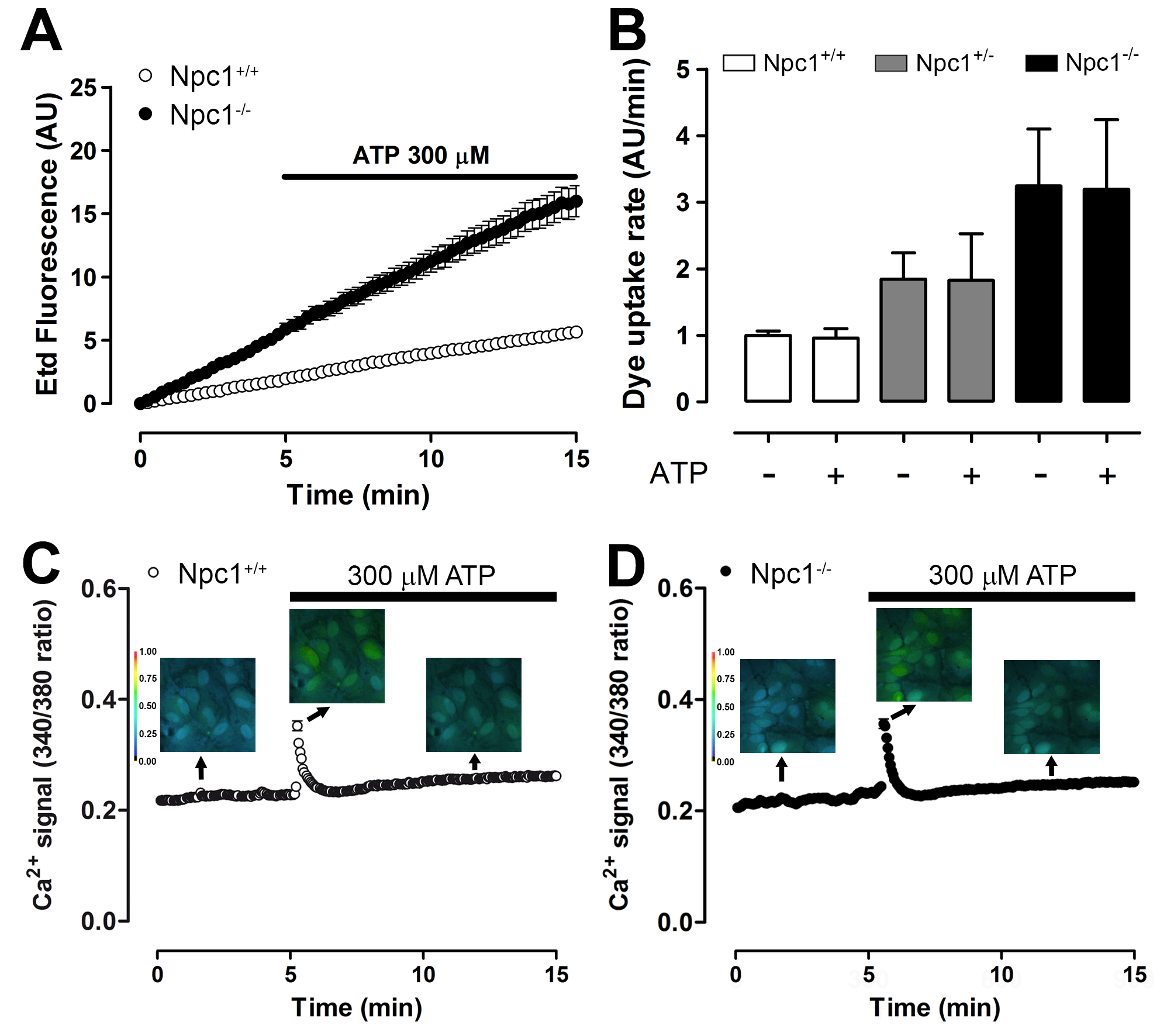

Supplement: Figure S1 — Npc1−/− astrocytes do not exhibit ATP-induced dye uptake. (A) Time-lapse measurements of Etd uptake in Npc1+/+ and Npc1−/− astrocytes (white and black circles, respectively) exposed to 300 µM ATP. (B) Averaged data of Etd uptake rate of Npc1+/+, Npc1+/− and Npc1−/− astrocytes (white, grey and back bars, respectively) under control conditions or treated with 300 µM ATP (acutely added during experiment). No significant differences were observed after ATP treatment. (C-D) Representative plots of relative changes in the Ca2+ signal (340/380 ratio) over time in Npc1+/+ astrocytes (C, white circles) and Npc1−/− astrocytes (D, black circles) under control conditions or after treatment with 300 µM ATP. In each panel, three photomicrographs of time-lapse images show changes in the Fura-2 ratio (pseudo-colored scale). Averaged data were obtained from at least three independent experiments. Each value corresponds to the mean ± S.E. of 20 cells in a representative of three experiments. (TIF) [file pone.0071361.s001.tif]

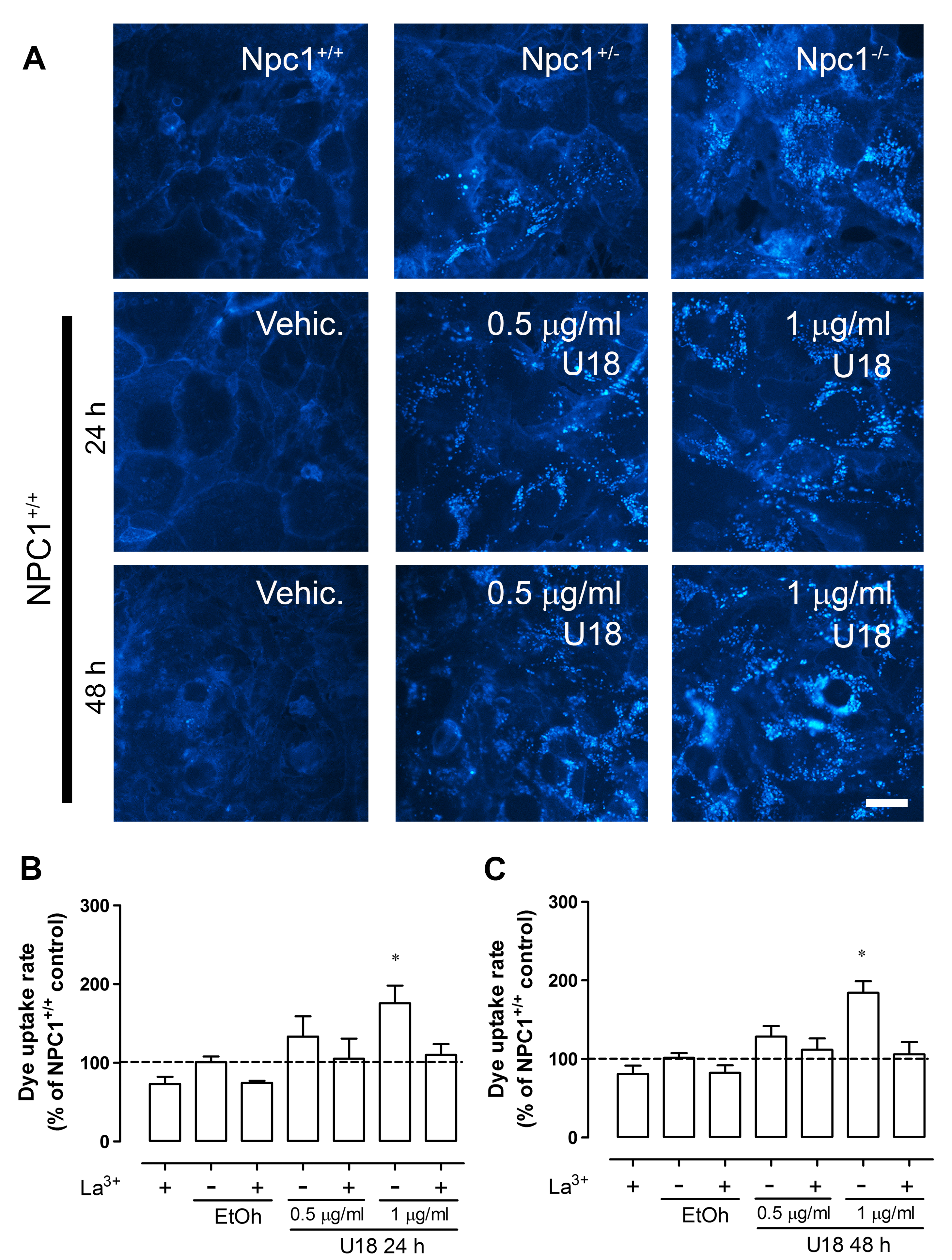

Supplement: Figure S2 — Cholesterol accumulation partially mimics the increased dye uptake of Npc1−/− astrocytes. (A) Fluorescent micrographs of filipin staining (blue) in Npc1+/+, Npc1+/− and Npc1−/− astrocytes. Also shown fluorescent micrographs of filipin staining in Npc1+/+ astrocytes exposed to vehicle (EtOh) or treated with 0.5 or 1 µg/ml U1866A for 24 or 48 h. Calibration bar = 25 µm. (B-C) Averaged data (normalized to control; dashed line) of the rate of Etd uptake by Npc1+/+ astrocytes exposed to vehicle or 0.5 and 1 µg/ml U1866A for 24 (B) or 48 h (C). Additionally, the effect of 200 µM La3+ applied acutely during Etd uptake experiments is shown. *p<0.05 compared to the basal level of Npc1+/+ astrocytes. The averaged data were obtained from four independent experiments. (TIF) [file pone.0071361.s002.tif]
